# Consensus methods in patellofemoral pain: how rigorous are they? A scoping review

**DOI:** 10.1136/bjsports-2023-107552

**Published:** 2024-05-22

**Authors:** Paul Blazey, Alex Scott, Clare L Ardern, Jennifer C Davis, Jackie L Whittaker, Justin M Losciale, Karim M Khan

**Affiliations:** 1 Department of Physical Therapy, Faculty of Medicine, The University of British Columbia, Vancouver, British Columbia, Canada; 2 Centre for Aging SMART at Vancouver Coastal Health, Vancouver, British Columbia, Canada; 3 Sport and Exercise Medicine Research Centre, La Trobe University, Melbourne, Victoria, Australia; 4 Applied Health Economics Laboratory, Faculty of Management, The University of British Columbia Okanagan, Kelowna, British Columbia, Canada; 5 Arthritis Research Canada, Vancouver, British Columbia, Canada; 6 Department of Family Practice, Faculty of Medicine, The University of British Columbia, Vancouver, British Columbia, Canada; 7 School of Kinesiology, The University of British Columbia, Vancouver, British Columbia, Canada

**Keywords:** Osteoarthritis, Consensus, Methods, Patellofemoral Pain Syndrome, Review

## Abstract

**Objective:**

Clinicians treating patients with patellofemoral pain (PFP) rely on consensus statements to make the best practice recommendations in the absence of definitive evidence on how to manage PFP. However, the methods used to generate and assess agreement for these recommendations have not been examined. Our objective was to map the methods used to generate consensus-based recommendations for PFP and apply four novel questions to assess the rigour of consensus development.

**Design:**

Scoping review.

**Data sources:**

We searched Medline, SPORTDiscus, CINAHL and Embase from inception to May 2022 to identify consensus-derived statements or practice guidelines on PFP. The Joanna Briggs Institute Manual for Evidence Synthesis was followed to map the existing evidence. We measured the consensus methods based on four sets of questions addressing the panel composition, application of the consensus method chosen, agreement process and the use of evidence mapping.

**Eligibility criteria:**

All consensus statements or clinical guidelines on PFP were considered.

**Results:**

Twenty-two PFP consensus statements were identified. Panel composition: 3 of the 22 (14%) consensus groups reported the panellists’ experience, 2 (9%) defined a desired level of expertise, 10 (45%) reported panellist sex and only 2 (9%) included a patient. Consensus method: 7 of 22 (32%) reported using an established method of consensus measurement/development. Agreement process: 10 of 22 (45%) reported their consensus threshold and 2 (9%) acknowledged dissenting opinions among the panel. Evidence mapping: 6 of 22 (27%) reported using systematic methods to identify relevant evidence gaps.

**Conclusions:**

PFP consensus panels have lacked diversity and excluded key partners including patients. Consensus statements on PFP frequently fail to use recognised consensus methods, rarely describe how ‘agreement’ was defined or measured and often neglect to use systematic methods to identify evidence gaps.

**WHAT IS ALREADY KNOWN?**

- Consensus statements aim to provide direction when evidence is not available, or when conflicts or interpretations of the evidence diverge. Currently, there is no standard method to evaluate the rigour of consensus statements.

**WHAT ARE THE NEW FINDINGS?**

- Published patellofemoral pain consensus statements have not used recognised methods to generate recommendations or assess agreement.
- Patellofemoral consensus processes have used a narrow definition of ‘expert’, seldom including ‘expertise’ outside of professional clinical experience. This has left key stakeholders, such as patients, under-represented and with a limited voice.
- Consensus panels have been male dominated and failed to include representatives from low or lower-middle income countries.
- Patellofemoral consensus statements often did not synthesize the evidence to identify knowledge gaps.

## Introduction

Consensus statements and their closely related cousins, position statements and clinical practice guidelines (herein referred to as ‘*statements*’), significantly influence clinical and research practices. Consensus methods are most often used by the scientific community to answer questions where scientific evidence is lacking, or when disagreements arise on the interpretation of the evidence.^1 2^ The employment of consensus methods and publication of their subsequent statements can direct large-scale research projects with significant implications for the future assessment and management of patients (for instance the Young Athlete’s Hip Research Collaborative or OPTIKNEE processes).^3–5^

Authors have criticised consensus processes for lacking methodological rigour, and neglecting to include all the key partners relevant to the problems they purport to address.^6–9^ This may call into question the authority of consensus statements and the utility of their recommendations.^10^

Expert agreement has often been sought on topics related to patellofemoral pain (PFP) due to evidence gaps, or a lack of knowledge/disagreement on how to apply what is known. For instance, the lack of definitive studies to inform the aetiology, prognosis and management of PFP, has necessitated the use of consensus methods to establish the best practice assessment and treatment, and to set research priorities.^11–13^ No previous study has mapped the methods used to gain consensus on topics related to PFP or patellofemoral osteoarthritis nor have the methods to generate recommendations and gain agreement been subject to scrutiny.^8^

Therefore, the objectives of this scoping review were to:

1. Map the consensus methods used to make practice recommendations on PFP or patellofemoral osteoarthritis.
2. Review the rigour of the methods using four novel questions related to: who was invited to participate; how consensus was generated; how subsequent agreement/dissent was reported; and whether scientific literature reviews were used to highlight gaps in the evidence, generate statements and/or inform panellist decisions.^10^

## Methods

This scoping review was conducted according to the Joanna Briggs Institute Manual for Evidence Synthesis,^14 15^ and reported following the Preferred Reporting Items for Systematic Reviews and Meta-Analyses Extension for Scoping Reviews (PRISMA-ScR) for reporting scoping reviews.^16^ The published protocol is available on the open science framework (https://osf.io/y2m3p/).

### Definitions

The only taxonomy of consensus-based studies that exist in the medical literature is from the European Cystic Fibrosis Society (ECFS, 2014).^17^ Building on the ECFS taxonomy, the following definitions were used for the purposes of this scoping review:

*Consensus statement*: a statement that results from a consensus generation process involving interested partners, which explicitly includes a voting process to measure level of agreement.

*Position statement*: a statement from a specific group(s) or party that may or may not include methods to generate consensus, nor an explicit voting process.

*Clinical practice guideline*: a report that may or may not include a rigorous systematic review and synthesis of the published medical literature.^18^ These may also involve a consensus process and a formal rating of the evidence (eg, using The Grading of Recommendations Assessment, Development and Evaluation (GRADE)).^19^

### Eligibility criteria

We included consensus statements, position statements or clinical practice guidelines (as described above) that provided recommendations on the assessment, diagnosis and/or management of PFP. Although there is some debate over whether PFP is a direct precursor to patellofemoral osteoarthritis (ie, that they exist on a continuum), we decided to include statements on patellofemoral osteoarthritis. Consensus was operationalised as a report that voting or another method of consensus generation among participants was used to arrive at a set of final reported recommendations. Examples of a clearly identified consensus methodology included the modified or unmodified Delphi, Nominal Group Technique, RAND-UCLA appropriateness method, or informal agreement among participants. Any report that identified as a ‘consensus statement’ was included for review, even in the absence of clear consensus methods.

We excluded reports of clinical practice guidelines that did not use a recognised consensus method—normally due to their reliance on evidence summaries such as GRADE—to reach their recommendations (eg, Willy *et al*, 2019—Patellofemoral Pain Clinical Practice Guidelines).^19^ Statements that focused on traumatic causes of PFP including patellofemoral instability post dislocation or PFP in the presence of hypermobility were also excluded.

### Information sources

To identify appropriate statements, the following bibliographic databases were searched: Medline (via Ovid); SPORTDiscus; CINAHL (via EBSCO); and Embase (via Ovid). All databases were searched from database inception to 4 May 2022. A medical research librarian supported the development of a comprehensive search strategy (see acknowledgements). An example of the full search strategy is presented for Medline (via Ovid) in table 1. The search strategies for all databases can be found in online supplemental appendices A1-A4.

10.1136/bjsports-2023-107552.supp1 Supplementary data

**Table 1:** Search strategy for Ovid Medline

Search number	Query	Results
1	Patellofemoral Pain Syndrome/	1082
2	(patellofemoral adj3 (pain or syndrome)).mp. [mp=title, abstract, original title, name of substance word, subject heading word, floating sub-heading word, keyword heading word, organism supplementary concept word, protocol supplementary concept word, rare disease supplementary concept word, unique identifier, synonyms]	2290
3	(patellar femoral adj3 (pain or syndrome)).mp. [mp=title, abstract, original title, name of substance word, subject heading word, floating sub-heading word, keyword heading word, organism supplementary concept word, protocol supplementary concept word, rare disease supplementary concept word, unique identifier, synonyms]	3
4	PFPS.mp.	533
5	anterior knee pain.mp.	2044
6	Chondromalacia Patellae/	96
7	Sinding larsen johansson.mp.	43
8	runner* knee.mp.	29
9	plica syndrome.mp.	119
10	1 or 2 or 3 or 4 or 5 or 6 or 7 or 8 or 9	4228
11	Consensus/	18 440
12	Consensus Development Conference/	12 306
13	Consensus Development Conference, NIH/	801
14	(Consensus adj3 (statement or paper)).mp. [mp=title, abstract, original title, name of substance word, subject heading word, floating sub-heading word, keyword heading word, organism supplementary concept word, protocol supplementary concept word, rare disease supplementary concept word, unique identifier, synonyms]	7476
15	((position or policy) adj3 (statement or paper)).mp. [mp=title, abstract, original title, name of substance word, subject heading word, floating sub-heading word, keyword heading word, organism supplementary concept word, protocol supplementary concept word, rare disease supplementary concept word, unique identifier, synonyms]	11 880
16	Practice Guideline/	29 792
17	practice guideline.mp.	35 320
18	Declaration.mp.	9682
19	11 or 12 or 13 or 14 or 15 or 16 or 17 or 18	84 269
20	10 and 19	14

All articles that met the inclusion criteria for full-text review underwent bibliometric indexing (backward citation tracking) of their references to search for references to previous consensus or position statements, or clinical practice guidelines on PFP. Where articles were not published in English, they were translated using Google Translate. No article was excluded due to language restrictions.

A comprehensive grey literature search was also developed in collaboration with the medical librarian, based on search guidelines from Godin *et al*.^20^ Briefly, this strategy involves four key themes: targeted website searching and browsing; grey literature database searches using sites such as Proquest Dissertations and Theses Global; search engine searches conducted in line with the best practice guidance offer by Haddaway *et al* 21; and contacting knowledge experts. Detailed explanation of all grey literature searches can be found in online supplemental appendix A5.

All searches were transferred into Covidence (Veritas Health Innovation). All titles and abstracts were screened by two reviewers (PB and JML). Articles that passed title and abstract screening were retrieved in full text to further gauge eligibility against the eligibility criteria. A pilot was conducted with three studies to ensure consistency between reviewers. Once calibration had taken place, all texts were read in full by both reviewers. Where disagreements occurred over inclusion in the final review, these were resolved via discussion and if necessary the vote of a third team member (KMK).

### Data charting

A data charting template was created to extract data from included studies. This was piloted with five studies (PB and JML) to ensure consistency in reporting or ranking items, as recommended best practice data extraction techniques for scoping reviews.^22^ Where information was not available, the contact authors for each source were contacted via email on at least two separate occasions to request further information.

Data extraction (see online supplemental appendix B for the full data charting template) included the following categories, divided into research metadata, and the primary and secondary aims.

#### Metadata

- Title.
- First author.
- Year published.
- Years since previous iteration (if applicable).
- Stated aim of the consensus process (examples include to derive treatment recommendations, or set priorities for future research).

#### Data extracted on consensus development process

- Number of panellists/experts.
- Experience of panellists (years).
- Definition of expertise (if present).
- Inclusion criteria for panellists (if present).
- Sex balance of the panel.
- Countries represented on the panel.
- Low/lower-middle income countries represented on the panel.
- Mix of partners (professions, patients, policy-makers) included.
- Whether a Stakeholder Analysis was completed.
- Whether questions were explicitly systematic or scoping review informed.
- Whether the questions asked of panelists were presented (either in the text or online supplemental material).
- What consensus method was reported (examples include Delphi, RAND-UCLA, Nominal Group Technique).
- Which method of consensus was used (if different from that reported in the methods or if no method stated then listed as ‘unclear’).
- Was the consensus level of agreement decided a priori (before the process began).
- What was the method used to represent agreement of the panel.
- Were dissenting opinions acknowledged and reported.
- Were funding/conflicts of interest reported.

Box 1 provides definitions to explain how we operationalised some of the criteria listed in the methods of consensus development.

Box 1 **Glossary of definitions**

*Definition of expertise*: would include any rationale supplied by the authors to explain why their panel qualified as ‘experts’ to answer the questions their process aimed to address.

*Sex-balance among panels*: the sex split of panels was estimated from given names reported in the final manuscript, or where unclear from web searches.

*Low or lower-middle income countries*: the involvement of representatives from low or lower-middle income countries was defined by noting the inclusion of at least one panel member from a country listed in either category by the World Bank (https://datahelpdesk.worldbank.org/knowledgebase/articles/906519-world-bank-country-and-lending-groups).

*Stakeholder analysis*: the use of a formal method to identify potential parties or partners that would be either interested or impacted by the statement, and therefore invited to participate in the process (including but not limited to: gaining consensus; approving the statement; implementation of the recommendations).

*Questions informed by systematic or scoping review*: is there a clear process for how the scientific review of available literature led to the questions presented to the panel either in the main statement or online supplemental material?

*Acknowledgement of dissent*: did the statement include any information on items that proved contentious among the panel? Simply saying an item was removed from agreement was not enough, there needed to be a clear discussion of what items may have been included despite a large number of votes against inclusion. Ideally with additional explanation as to why.

### Critical appraisal: using a novel tool to assess methodological rigour

Our original protocol outlined data charting, but no process of appraisal. Scoping reviews have been criticised for not including a quality assessment, which makes interpretation of the data challenging.^23^ In a deviation from our protocol (https://osf.io/y2m3p/), we decided to perform a qualitative content analysis.^22^

There is currently no known quality-rating system with which to design or judge consensus-based methods, and the reporting guideline for consensus-based methods in biomedical research was published following the completion of our work.^24 25^ Therefore, in the absence of a reporting or quality guideline with which to describe or assess a consensus development process and its subsequent statement, we used four sets of questions as a lens through which to view existing statements.^10^ These four sets of questions were previously described as supporting an evidence-informed appraisal of the conduct of consensus development in sport and exercise medicine.^10^ Critically, the four sets of questions were based on both the Conducting and Reporting Delphi Studies guideline and critiques from the literature on consensus development processes.^26^

**Figure 1:**
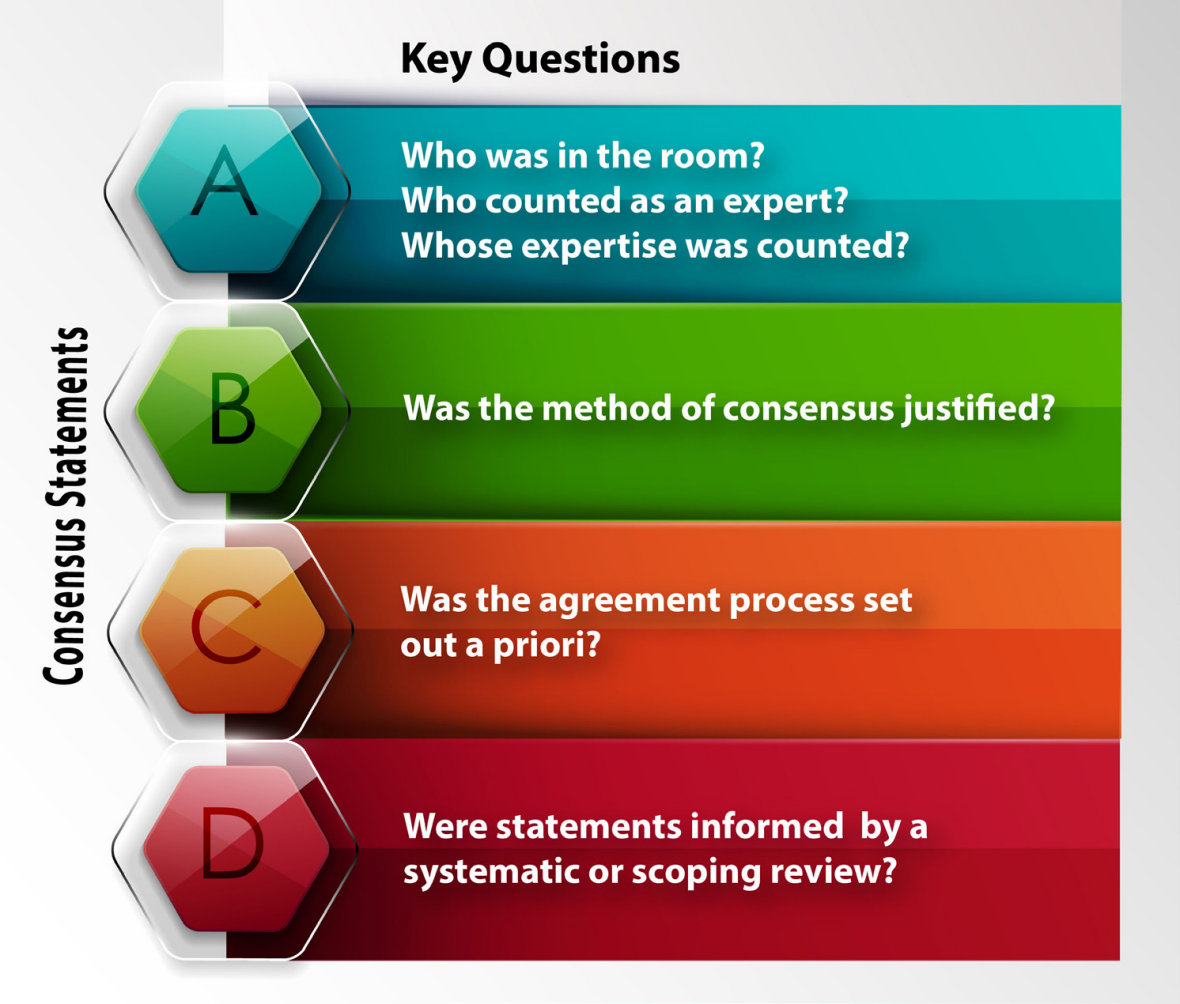
Four sets of questions that support the assessment of rigour during consensus development.

The four sets of questions that were used to frame existing consensus development processes are outlined as A–D in figure 1.

#### Synthesis of the results

Data are grouped into both narrative summaries and summary tables of the extracted data. Part A presents the data on participants on the consensus panel or steering committee including:

- panel number;
- panel expertise/experience;
- inclusion criteria for panellists;
- sex split of panels;
- and participant groups represented.

Part B focuses on the method and justification for reaching consensus.

Part C focuses on the individual procedures identified for observing when consensus was achieved including:

- was consensus operationally defined a priori;
- what was the level of agreement (expressed either as a percentage or categorical measure);
- and were dissenting opinions acknowledged in the final report.

Finally, part D looks at the methods for generating questions or providing information to the panel. This included description of whether a systematic or scoping review was performed prior to the consensus process, and whether the questions asked were explicitly reported.

All items were tabulated using Microsoft Excel.

### Patient and public involvement

No patients were involved in the development of this review.

### Equity, diversity and inclusion statement

The authorship group consists of early, mid and late-career researchers and clinician scientists inclusive of a Master’s student, PhD candidate, assistant, associate and full professor. The researchers or clinician–researchers originate from the UK, Canada, the USA and Australia. Five are registered physiotherapists, one sport and exercise medicine specialist, and one professor of health economics. The authors are 43% female, and 86% identify as white.

This is a synthesis of existing research but the results focus on sex balance, patient and professional representation and the representation on consensus panels of those from low or lower-middle income countries (with crossover between income status as defined by the World Bank, and nations considered part of the ‘Global South’). Our study considered diversity as a marker of rigourous and representative consensus development. It is possible (hopeful) that the results of this work will inform future consensus processes and encourage the inclusion of members from more diverse and representative backgrounds.

## Results

### Selection of sources of evidence

**Figure 2:**
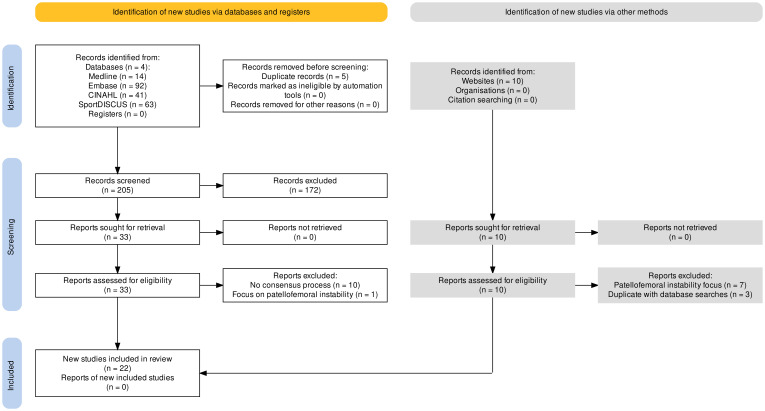
Preferred Reporting Items for Systematic Reviews and Meta-Analyses flow chart of returned searches.^78^

Figure 2 shows the PRISMA flow chart of evidence management. We identified 225 records. After title/abstract screening, 33 records were screened at full-text and 22 articles were included. Online supplemental appendix A contains the database and grey literature search results.

### Characteristics of sources of evidence

Table 2 provides an overview of the included statement’s characteristics. Of the 22 statements included, 15 focused directly on PFP, and 7 included at least 1 statement on PFP (or patellofemoral osteoarthritis). Consensus statements on PFP have become more popular with four published in each of 2018 and 2021. The aims of the consensus processes have been heterogenous. The majority (13%–59%) have looked to establish agreement on treatments or interventions related to PFP. Other aims have included: definitions—1 (5%); diagnosis—5 (23%); natural history of PFP—5 (23%); agree on patient-reported outcome measures—2 (9%); a reporting checklist for PFP studies—1 (5%); and priority setting for research related to PFP—2 (9%).

**Table 2:** Characteristics of the included statements (see online supplemental material for detailed version of table 2)

First author	Year published	Stated aim (eg, treatment recommendation, develop definitions, priority setting exercise, etc)
Herring^27^	2008	Help the team physician improve the care of the adolescent athlete by understanding the medical, musculoskeletal (shoulder/knee—including patellofemoral pain (PFP)/elbow/spine), and psychological issues (sport specialisation) common in this age group
Davis^11^	2010	Unclear—presentation of research and some future research recommendations
Powers^79^	2012	To understand the factors that contribute to the development and, consequently the treatment of PFP
Witvrouw^80^	2014	Gain consensus for three specific areas: (1) the natural history of PFP and local (knee region) factors that influence PFP; (2) trunk and distal factors that influence PFP; and (3) innovations in rehabilitation for PFP
McAlindon^31^	2014	To develop concise, up-to-date, patient-focused, evidence-based, expert consensus guidelines for the management of knee osteoarthritis (OA) *including patellofemoral osteoarthritis*
Crossley^71^	2016a	To agree on: terminology, definitions, clinical examination, natural history and PROMs (patient-reported outcome measures)
Crossley^39^	2016b	To agree on expert-recommended physical interventions for PFP
Herring^28^	2016	To help the team physician improve the care of the athlete by understanding and practising methods of injury and illness prevention in specific sports medicine problems.
Powers^81^	2017	To place known associated factors within the context of a pathomechanical model of PFP
Herring^29^	2018	To improve the care of the female athletes by understanding select injuries and illnesses
Van Middlekoop^36^	2018	Mixed consensus on: diagnosis; burden; PROMs; prognosis; risk factors; and treatment. Adds in a priority-setting process.
Collins^37^	2018	To agree on expert-recommended physical interventions for PFP
Huang^82^	2018	To formulate Chinese Pain Specialist Consensus on the diagnosis and treatment of degenerative knee osteoarthritis (DKOA)
Fox^30^	2018	To provide clinicians with the best practices for ordering imaging examinations
Guanghua^83^	2020	Guidelines on the diagnosis and treatment of patellofemoral OA
Chahla^32^	2020	Consensus on the functional anatomy, indications, donor graft considerations, surgical treatment and rehabilitation of large chondral and osteochondral defects in the patellofemoral joint
Kolasinski^38^	2020	To develop an evidence-based guideline for the comprehensive management of osteoarthritis (OA) as a collaboration between the American College of Rheumatology and the Arthritis Foundation, updating the 2012 ACR recommendations for the management of hand, hip and knee OA.
Keshmiri^84^	2021	Consensus on therapy for different patellofemoral abnormalities in patients suffering from isolated patellofemoral arthritis.
Kunene^33^	2021	To develop a community-based rehabilitation implementation framework for PFP in runners from under-resourced communities
Barton^34^	2021	Consensus statement and associated checklist provide standards for REPORTing of quantitative PatelloFemoral Pain (REPORT-PFP) research to enhance clinical translation and evidence synthesis, and support clinician engagement with research and data collection
Guanghua^85^	2021	To summarise the latest research progress in the surgical treatment of patellofemoral osteoarthritis, and refer to the latest domestic and foreign guidelines and consensus
Vicenzino^35^	2022	The objective of this consensus development process was to decide clinical and research priorities for pain features and psychological factors in persons with PFP.

ACR, American College of Rheumatology.

10.1136/bjsports-2023-107552.supp2 Supplementary data

### Synthesis and appraisal of results

#### Representativeness of PFP statement panels (part A)

Table 3 provides detail on the representativeness of panels. The number of panellists included ranged from 10 to 71. Only 3 (14%) of the 22 reports detailed the experience of their respective panels, and only 2 (9%) of these 3 gave further details as to how they defined expertise prior to recruiting their panellists. Eight (36%) studies provided inclusion criteria for the selection of their panellists. Four (18%) reports had existing criteria for panellist selection detailed on linked websites.^27–30^ Five (23%) studies outlined their own individual methods for highlighting experts.^31–35^ Five (23%) were classed as ‘unclear’ because they reported panellists had to have been part of a recent meeting related to the topic under discussions without providing qualifying criteria as to why presence at the meeting made the panellists suitable.

**Table 3:** Representativeness of patellofemoral pain (PFP) statement panels (see online supplemental material for detailed version of table 3)

First author	No. panellists	Definition of expertise	Inclusion criteria for panellists	Sex split	No countries represented
Herring (2008)	11	Not applicable	Yes*	7 male: 4 female	1—USA
Davis (2010)	Unclear	Not applicable	Unclear	Unclear	10
Powers (2012)	Unclear	Not applicable	Unclear	Unclear	9
Witvrouw (2014)	Not reported	Not applicable	Unclear	Unclear	Not reported
McAlindon (2014)	13	Not applicable	Yes	10 male: 3 female	10
Crossley (2016a)	Not reported	Not applicable	No	Unclear	Not reported
Crossley (2016b)	35	Not applicable	Unclear	Unclear	Not reported
Herring (2016)	12	Not applicable	Yes*	10 male: 2 female	1—USA
Powers (2017)	Not reported	Not applicable	Unclear	Unclear	Not reported
Herring (2018)	10	Not applicable	Yes*	4 male: 6 female	2—USA and Canada
Van Middlekoop (2018)	Unclear	Not applicable	Unclear	*Authorship* 8 male : 9 female	Unclear
Collins (2018)	41	‘active researchers in the field’	Unclear	*Authorship* 6 male: 6 female	Unclear
Huang (2018)	15	Not applicable	No	Unclear	1—China
Fox (2018)	17	Not applicable	Yes†	8 male: 9 female	1—USA
Guanghua (2020)	30	Not applicable	No	Unclear	1—China
Chahla (2020)	28	Not applicable	Yes	26 male: 2 female	2—USA and Canada
Kolasinski (2020)	15	Not applicable	No.	8 male: 7 female	USA and Canada
Keshmiri (2021)	13	Not applicable	No.	12 male: 1 female	3—Germany, Austria and Switzerland
Kunene (2021)	19	> 5 years post qualification	Yes.	10 female: 9 male	2—South Africa and UK
Barton (2021)	24 (2015); 51 (2019)	Measured in clinical experience	Yes.	Unclear*	10 countries
Guanghua (2021)	35	Not applicable	No	Unclear	1—China
Vicenzino (2022)	Survey: 35 healthcare workers and 36 patients In-person: 20 healthcare workers	Years of experience or exposure to patients with PFP	Yes	Survey: healthcare workers (20 male:15 female); and patients (12 male:24 female) In-person: (11 male: 9 female)	9—health professions survey 4—patient survey 8—in-person process

*Panel is made up from two nominated representatives from each of American Academy of Family Physicians, American Academy of Orthopaedic Surgeons, American College of Sports Medicine, American Medical Society for Sports Medicine, American Orthopaedic Society for Sports Medicine and American Osteopathic Academy of Sports Medicine. Representatives are chosen by their organisation based on their experience as team physicians with expertise in the topic area.

† Available on American College of Radiology website—‘Following regulatory requirements, we survey panel members on their skills and expertise to ensure that panels include expertise in the clinical topic, primary care medicine, medical imaging, statistics and clinical trial design. Panel members’ expertise is determined using self-attestation and calculated by the amount of education, training and experience the member reports for that skill area’.

10.1136/bjsports-2023-107552.supp3 Supplementary data

One statement explicitly reported participant sex,^35^ and one reported panellists preferred gender identity.^33^ Ten (45%) studies included enough information on panellists or authors for us to estimate their sex on the basis of names and/or internet profile data. Of the 10 articles, 8 (80%) had greater male representation than female, with the greatest difference being a 26:2 male:female panel.^32^ There were two further studies where the panel size had a large discrepancy from the authorship; in these instances, we collected the estimated sex of the authors. One authorship team had greater female representation than male (9:8),^36^ and one authorship was balanced (6:6).^37^

Countries represented on the panels ranged from 1 to 10, with 16 (73%) statements appearing to be based on the opinions of multicountry panels. The USA was the most commonly represented country with clear indications that panellists or authors originated from the USA in 16 (73%) of the statements. Only one consensus statement—Barton *et al* (2021)—included a panellist where a member was considered to be from a low or lower-middle income country (India).

Thirteen of the 22 (59%) articles detailed the professional designations of their panellists. The most commonly represented professions invited to provide statements on topics related to PFP were medical doctors of no known specialty (n=11%–50%), orthopaedic surgeons or specialists (n=11%–50%) and physiotherapists (n=8%–36%). Patients were part of the panel in two studies (9%).^31 38^ Vicenzino *et al*. (2022) did include patients at the survey stage of their development process to support clinical decisions, but patients were not invited to be part of the final decision-making process.

Four statements (18%) clearly reported any conflicts of interest among invited panellists. Four further studies (18%) included either a statement declaring authors had no conflicts of interest or where funding had been given to generate the statement. This left 14 articles (64%) without either a conflict of interest statement, or a disclosure of any funding received.

#### Method of assessing/achieving consensus and definition of consensus (parts B and C)

Table 4 details the methods used for measuring and/or facilitating consensus on PFP. Seven (32%) articles reported an identified method of consensus to elucidate their panellists’ views (five Delphi, and two RAND-UCLA technique). A further three studies reported their own methods (two scale-based and one survey plus in-person). Nine had no identifiable method, and three were unclear.

**Table 4:** Methods used for measuring, and/or facilitating consensus among panel member (see online supplemental material for detailed version of table 4)

First author	What consensus method was reported?	Which method of consensus was used?	Was consensus level decided a priori?	What was the method or level of agreement set at?	Were dissenting opinions acknowledged and reported?
Herring (2008)	None	Informal—iterative development over written rounds and in-person meeting	No	Unanimous agreement among invited panel—assumed *not* measured	No
Davis (2010)	None	Consensus conference—informal	No	Unclear	No
Powers (2012)	None	Consensus conference—informal	No	Unclear	No
Witvrouw (2014)	None	Consensus conference—informal	No	Unclear	No
McAlindon (2014)	RAND-UCLA and Delphi	RAND-UCLA	Yes	RAND method	Yes
Crossley (2016a)	None	Consensus conference—informal	No	Unclear	No
Crossley (2016b)	Unclear	Modified version of RAND/UCLA	Yes	Had to be rated 'appropriate' (7–9 on a 10-point Likert) on average (median) *AND* consistent with evidence	No
Herring (2016)	None	Informal—iterative development over written rounds and in-person meeting	No	Unanimous agreement among invited panel—assumed *not* measured	No
Powers (2017)	None	Informal—not reported fully	No	Unclear	No
Herring (2018)	None	Informal—iterative development over written rounds and in-person meeting	No	Unanimous agreement among invited panel - assumed *not* measured	No
Van Middlekoop (2018)	Numerical Rating Scale 0–10	Unclear	Yes	For priority setting part only, consensus was >7.5 out of 10 on numerical rating scale	No
Collins (2018)	10 point Likert scale—median score must be between 7 and 9	Unclear	Yes	Median agreement between 7 and 9	No
Huang (2018)	None	Unclear	Unclear	Unclear	No
Fox (2018)	RAND-UCLA	RAND-UCLA	Yes	median agreement between 7 and 9	Yes
Guanghua (2020)	Delphi	Delphi	Unclear	Unclear	No
Chahla (2020)	Delphi	Modified Delphi	Yes	Over 75% of respondents agreed and fewer than 20% disagreed in the final voting round	No
Kolasinski (2020)	Unclear	Unclear	Yes	>70% agreement	No
Keshmiri (2021)	Delphi	Modified Delphi	No	Unclear	No
Kunene (2021)	Delphi	Modified—Delphi	Yes	>70% agreement	No
Barton (2021)	Delphi	Modified Delphi mixed with priority setting process	Yes	>70% agreement	No
Guanghua (2021)	Unclear	Unclear	Unclear	Unclear	No
Vicenzino (2022)	Survey plus in person meeting	Survey plus in-person meeting	Yes	>70% agreement on survey and paper-based votes (single round)	No

10.1136/bjsports-2023-107552.supp4 Supplementary data

Qualitative assessment revealed substantial deviations from the reported method in all but two studies.^30 35^ Many of the articles that did not report a method used either an informal process of developing a written document over successive editing rounds without a formal voting structure (authors’ signing off at the end of the process)—sometimes called ‘Glaser’s State-of-the-Art Approach’,^6^ or used a form of consensus conference to generate statements which were taken away by a small group to be written up. Many of those who reported using a Delphi method used a modified Delphi with an in-person element to decide on final statements.

Ten (45%) articles reported deciding on what was considered consensus among panellists a priori. Of these, four studies fixed consensus as meaning 70% of panellists agreed with the statements. Three used a derivation of the RAND-UCLA criteria with the mean among panellists falling within the 7–9 range on a 9-point Likert scale when 9 was full agreement (one used a 10-point). One article^39^ reported that the median rank of ‘appropriate’ (using a 10-point Likert where agreement was a median score between 7–9 on a 0–9 scale) but final statements had to be in agreement with objective evidence from literature searches.^39^ It was unclear how (or who) this was decided by. One study^32^ set criteria that 75% had to agree with a statement while no more than 20% could disagree on a 5-point Likert scale where 4 and 5 were agree/strongly agree.^32^ One study^36^ did not explain how statements were voted on or agreed on among panellists, but did report the results of consensus on subsequent research priorities (numerical scale 0–10, with consensus set at>7.5).^36^

Two of the 22 (9%) articles reported on dissenting opinions. Both consensus processes used the RAND-UCLA technique where dissent is expressed as part of the traditional quantitative assessment. No report explored the meaning of any expressed dissent among panellists.

#### Use of scientific literature searches to support question formation or delegate decision-making and conflicts of interest (part D)

Six of the 22 (27%) articles reported using systematic methods to inform the statements used in their consensus development.^30 31 35 37–39^ Four of the six^30 31 35 38^ provided links to their systematic searches and/or summaries of the evidence which were given to panel members to support decisions made during the consensus process. One further article reported a partial literature review, and three reported informal literature reviews, with no supporting information provided.

Eight of the 22 (36%) articles explicitly recorded the questions that panellists were asked to vote on. Table 5 summarises which consensus processes used literature searches, whether they reported the search results, and whether or not they made the questions that were produced by said searches explicit in their reports or the supplementary material.

**Table 5:** Methods informed by appropriate systematic or scoping review

First author	Were questions informed by a systematic or scoping review?	Searches and information summary in the report or online?	Were the questions asked of panellists made explicit?
Herring (2008)	No	N/A	No
Davis (2010)	No	N/A	No
Powers (2012)	No	N/A	No
Witvrouw (2014)	No	N/A	No
McAlindon (2014)	Yes	Yes	Yes
Crossley (2016a)	Partial literature review of natural history (PFP and patellofemoral osteoarthritis) and patient reported outcome measures for PFP	No	No
Crossley (2016b)	Yes	No	Yes
Herring (2016)	No	N/A	No
Powers (2017)	No	N/A	No
Herring (2018)	No	N/A	No
Van Middlekoop (2018)	No	N/A	No
Collins (2018)	Yes	No	Yes
Huang (2018)	No	N/A	No
Fox (2018)	Yes	Yes	Yes
Guanghua (2020)	No	N/A	No
Chahla (2020)	No	N/A	Yes
Kolasinski (2020)	Yes	Yes	No
Keshmiri (2021)	No	N/A	Yes
Kunene (2021)	No	N/A	No
Barton (2021)	No	N/A	Yes
Guanghua (2021)	No	N/A	No
Vicenzino (2022)	Yes	Yes	Yes

PFP, patellofemoral pain.

## Discussion

Consensus methods have evolved over the past 70 years. The most common methods include Delphi outlined in the 1950s^40 41^; Nominal Group Technique originating in the 1970s^42 43^ and the RAND-UCLA method developed in the early 1990s.^44^ Choosing to bypass these recognised methods of consensus development is not necessarily a weakness when there is a clear rationale for that decision.^25^ Authors should pick the methods that best suit their aims and fit with the resources available to them. It is logical that there is heterogeneity among the approaches groups choose to generate consensus. We found that consensus seekers in PFP or patellofemoral osteoarthritis chose recognised methods of consensus development (eg, the Delphi method or RAND-UCLA appropriateness method) less often (32% of statements) than consensus statements in some other areas of medicine. For instance, Delphi or modified Delphi was used in 196 out of 257 (76%) of consensus approaches to medical education topics between 2009 and 2016.^8^

Our review found that many consensus statements on PFP (or patellofemoral osteoarthritis) published between 2008 and 2022 missed steps that support the rigorous development of consensus recommendations.^10 45 46^ However, we acknowledge that the framework we used to evaluate rigour was published in 2021 and has not been validated. Our use of the four questions outlined in figure 1 to interrogate the rigour of past consensus processes will, we hope, increase researchers’ awareness of key questions to consider.

Bearing in mind the historical context in which some of the existing consensus statements were conducted, we used four sets of questions to evaluate the rigour of existing PFP consensus development. We found that most consensus statements failed to address at least one of the four key areas. These four areas constitute: panel representation and diversity; using recognised methods of consensus development; defining what constituted ‘agreement’; and/or appraising literature to identify knowledge gaps.

### Panel representation and diversity (part A—who was in the room? Who was counted as an ‘expert’? Whose ‘expertise’ counted?)

To obtain a clear and useful answer from a consensus panel, it is important that invited panellists are both knowledgeable, and representative of the population the answers will serve.^47^ The panellists recruited to develop consensus on topics related to PFP have been: male dominated (80%); largely from high income countries (especially North America—USA or Canada represented in 73% of panels, Western Europe—52% and Australia—43%); and, without justification, focus on medical doctors, allied health professionals and researchers. Low or lower-middle income countries were represented in only one consensus panel (5%). Patients have largely been absent—only two statements included a patient on their panel. Questions on diagnosis and treatment (ie, those most concerning patients) were the most commonly asked in the PFP/patellofemoral osteoarthritis consensus-based research, and therefore it might have been expected that patients would be more involved.

In some cases, it may be appropriate for consensus panels to focus on ‘experts-only’.^48 49^ The recently developed reporting guideline for consensus exercises recommends detailed reporting of the criteria for panellist inclusion.^2 25^ We note that most consensus developers did not provide definitions of expertise other than ‘experience’. Expertise and experience are conceptually different and we encourage deeper consideration of the use of ‘expertise’ to justify the make-up of consensus panels. Too much group homogeneity may lead to a lack of critical questioning among the panel, or panellists not being able to recognise potential conflicts of interest.^47 50–52^ The narrow definition of ‘expert’ and exclusion of patients also ignores the ethical consideration of patients being integral to decisions made about their care.^53^ No PFP statements thus far have used stakeholder analysis or engagement theories to select their panels.^54–58^ We propose that a lack of key group involvement in decision-making processes could harm subsequent implementation of recommendations.

### Using recognised methods and defining consensus (parts B ‘was the method of consensus justified?’ and C ‘was the agreement process set out a priori?’)

Fewer than half (32%) of the statements on PFP used identifiable methods of consensus development. Failing to use a formal method runs the risk that consensus seekers will miss the steps associated with rigorous scientific research.^59^ Although consensus is iterative, it should also be guided by a framework, without which there is a risk that decisions are made based on individual (potentially biased) opinions.^6 60 61^

Two (9%)^30 31^ of the included studies did have rigorous methodology underpinning their statements, having identified and used the RAND-UCLA appropriateness method which has an extensive open-access guide available at https://www.rand.org/pubs/monograph_reports/MR1269.html.^44^ The organisations (Osteoarthritis Research Society International, and the American College of Radiology) supporting statements that used the RAND-UCLA appropriateness method also had extensive supporting literature detailing their processes for arriving at their statements, how they selected panellists, and consistently applied these criteria across several other consensus statements on topics not eligible within this review.

Fewer than half (45%) of the consensus statements developers used a predefined threshold to establish when agreement existed among their panel. Failing to define agreement can lead to prolonged processes or premature declarations of agreement among panellists in the absence of unanimity.^62^ However, it has to be acknowledged that there is no gold standard for measuring when agreement exists among a group. There were several statements where no apparent vote was used. Implicit agreement among a panel is potentially misleading, and may be a result of people feeling they have not been given a platform to voice opinions. This runs the risk that those with the greatest power (loudest voice) will dominate such proceedings.^42 60 63 64^

Only two studies reported the presence of dissent among their panel.^30 31^ Both consensus-based studies that acknowledged dissent used the RAND-UCLA method. However, neither study formally explored the reasons for the dissenting opinions among their respective panels. Not acknowledging disagreement (and the reasons behind disagreement or dissent) may seem normal in statements that report on agreement, but risks suppressing relevant counteropinions.^9 10^ Groups that are forced to agree run the risk of agreeing to watered-down statements.^65^ Suppression of minority opinions is just one of the reasons the Concussion in Sport Group was criticised for their statements on concussion in sport.^7^

### Appraising literature and identifying knowledge gaps (part D—‘Were statements informed by a systematic or scoping review?’)

Consensus is often used to arrive at statements (or guidance) on topics when evidence is lacking, or to help integrate the available evidence into clinical practice.^2 10 66 67^ If there is no review of the existing evidence, it is hard to judge what consensus judgements should be focused on. Around a third of guidelines (34%) have been criticised for lacking systematic methods to synthesise information, and underpin their recommendations.^68^ Scoping reviews can generate valuable evidence ‘gap maps’.^15 69^ Previous critiques have already recommended that systematic literature synthesis be integrated into consensus methods.^66 70^

Only six (27%)^30 31 35 37 38 71^ of the statements on PFP or patellofemoral osteoarthritis explicitly reported using a formal review of the evidence to either guide statement formation, or to inform panellist decisions in the subsequent consensus process. Five out of the six statements using a formal review reported the questions their panellists were asked to generate recommendations explicitly, either within their manuscript or as online supplemental material.

Systematic searches can be used to form statements (which the consensus panel vote on), and/or to produce evidence summaries for panellists before they vote in a consensus process. No formal guidance exists on how to translate systematic literature searches into unbiased statements. Transparent and well-reported consensus statements should include all the material that was used to inform decisions made in the consensus process (often as online supplemental material).^25^

### Limitations

As yet no quality framework exists to judge consensus statements, and the reporting guideline (Accurate Consensus Reporting Document—ACCORD) was published in January 2024.^25^ The four sets of questions used to frame the consensus development processes in this study were derived from previous critiques of the consensus literature.^8 10 61 62 72 73^ These questions provide a means to view the data in this scoping review, but are not designed as a comprehensive quality assessment tool. Scoping reviews should not be used to evaluate the quality of existing evidence.^22^ The four sets of questions we used to frame our report on the rigour of consensus development here have not been validated. It is possible we missed questions that may have enhanced our understanding of the rigour of consensus development in statements reporting on PFP or patellofemoral osteoarthritis.

To assess the number of countries represented, we used panellists’ self-reported affiliations. This ignores the regular movement of people between countries. Panellists do not ‘lose’ their experiences or ‘knowledge’ of their countries of origin. It is possible that some of those counted among high-income countries originated from low-income or middle-income countries. Panellists who originated from low-income or middle-income countries may have brought valuable additional insights to their consensus processes that were not captured.

There are flaws in using conventional names to estimate the balance of sex or gender on panels. We consciously decided to report our data using sex and not gender, as sex provides a binary model (male vs female), as opposed to the spectrum of gender diversity. We do not wish to inadvertently misgender the panellists. We believed that we were less likely to mistake sex based on naming conventions and tried to coordinate our data using publicly available information on panellists or statement authors. We acknowledge that there may be errors where we have made assumptions. Automated tools have been used to assess gender balance in research reports but these suffer from only being able to produce binary reports, for example, https://genderize.io/ or https://namsor.app/about-us. The 2020 Elsevier report on gender in science which used the NamSor application to assert gender balance in research reported precision rates of 93% for men and 98% for women.^74^ For consensus panels to meet diversity and inclusion criteria, it would be useful for all future consensus projects to ask panellists their gender to facilitate clear and accurate reporting of the genders represented.

This review highlights the lack of key representative groups being included in consensus processes. However, and with regret, we—the authors—recognise our own failure to include a patient partner in this research project. While stating the need for diversity in consensus processes, we also recognise the lack of diversity among the authors. In hindsight, we feel adding patients and a more diverse steering committee would have added richness to our appraisal, especially with regards to our assessment of diversity, representation and expertise.

### Future directions

Future consensus statements on PFP should focus on developing representative panels to enhance creativity, and avoid the problems associated with ‘groupthink’. Sex and gender diversity among panels improves group decision-making, and thus this analysis, although crude, may still help to increase awareness among consensus seekers that panel memberships need to be diverse.^50 52 75 76^ Stakeholder analysis might form an innovative and objective way to develop future panels who represent all of those who might be impacted by the aims of a consensus exercise in PFP, or other topics in sports and exercise medicine. Consensus organisers could consider adopting the ‘7Ps Framework to Identify Stakeholders in Patient-Centered Outcomes Research’ where stakeholders are broken down into seven key groups: Patients and the public; Providers; Purchasers; Payers; Policy-makers; Product makers; and Principle investigators.^55^

Statements often reported involving clinician–researchers; if these panellists were predominantly research based, it could have affected the adoption of recommendations in clinical practice.^77^ Therefore, future statements should consider involving those actively practising with patients. Systematic or scoping reviews should be used to analyse gaps in existing literature, and guide consensus development panels on where their efforts should be directed.

This review framed existing consensus statements against questions on the rigour of consensus development. We did not assess whether consensus developers had begun to answer these four questions more often in more recently published work (ie, whether there was a time trend among published consensus statements). Future studies could assess whether consensus development methods are improving to inform what future actions may be needed to enhance the rigour of future consensus-based approaches.

Future assessments of quality should focus on the quality of consensus development methods (eg, effective use of Delphi, RAND-UCLA) and not the subsequent statements or recommendations of the consensus panel. The quality (accuracy) of the statement recommendations only becomes apparent over time and should evolve as new evidence and clinical solutions emerge. As a result, trust in consensus statements relies on the rigour of methods used to develop recommendations and agreement, and from the inclusion of diverse and representative panel members.

## Conclusion

Clinicians and researchers have sought consensus with increasing frequency on topics related to PFP. However, consensus statements on PFP have often failed to rigorously develop consensus recommendations with respect to the four questions we outlined in this review. The lack of systematic searching to identify potential evidence gaps may have resulted in statements focusing on areas with well-established research evidence, or missing important topics where no information exists. Given the potential for consensus to direct whole bodies of research, it is perhaps most concerning that the patient voice has been almost totally absent.

Future consensus statements that are rigorous, representative (of all interested or impacted parties) and clearly report their development processes could be seen as more credible.
