# Supplementary material for: Consensus methods in patellofemoral pain: how rigorous are they? A scoping review
Source: Br J Sports Med. 2024 May 22;58(13):733–44. doi: 10.1136/bjsports-2023-107552 (PMC11228197; doi:10.1136/bjsports-2023-107552)
Supplement: Supplementary data [file bjsports-2023-107552supp001.pdf]

Appendices

Appendix A - Literature search

A.1 Searches for Embase

Embase <1974 to 2022 May 3>

1	patellofemoral pain syndrome/	1646
2	(patellofemoral adj3 pain*).mp. [mp=title, abstract, heading word, drug trade name, original title, device manufacturer, drug manufacturer, device trade name, keyword heading word, floating subheading word, candidate term word]	2895
3	(patellar femoral adj3 pain).mp. [mp=title, abstract, heading word, drug trade name, original title, device manufacturer, drug manufacturer, device trade name, keyword heading word, floating subheading word, candidate term word]	7
4	PFPS.mp.	677
5	Anterior knee pain.mp.	2520
6	patella chondromalacia/	827
7	chondromalacia patellae.mp.	374
8	Sinding larsen johansson.mp.	79
9	runners knee.mp.	33
10	plica syndrome.mp.	168
11	1 or 2 or 3 or 4 or 5 or 6 or 7 or 8 or 9 or 10	6018
12	consensus/ or consensus development/	111133
13	(consensus adj3 (statement or guideline)).mp. [mp=title, abstract, heading word, drug trade name, original title, device manufacturer, drug manufacturer, device	10677

	trade name, keyword heading word, floating subheading word, candidate term word]	
14	(position adj3 (statement or guideline)).mp. [mp=title, abstract, heading word, drug trade name, original title, device manufacturer, drug manufacturer, device trade name, keyword heading word, floating subheading word, candidate term word]	5297
15	practice guideline/	495491
16	clinical practice guideline.mp.	6414
17	*health care policy/	71057
18	policy paper.mp.	154
19	12 or 13 or 14 or 15 or 16 or 17 or 18	665891
20	11 and 19	92

A.2 Search strategy for Medline via Ovid

Ovid MEDLINE(R) and Epub Ahead of Print, In-Process, In-Data-Review & Other Non-Indexed Citations, Daily and Versions <1946 to May 04, 2022>

1	Patellofemoral Pain Syndrome/	**1082**
2	(patellofemoral adj3 (pain or syndrome)).mp. [mp=title, abstract, original title, name of substance word, subject heading word, floating sub-heading word, keyword heading word, organism supplementary concept word, protocol supplementary concept word, rare disease supplementary concept word, unique identifier, synonyms]	**2290**
3	(patellar femoral adj3 (pain or syndrome)).mp. [mp=title, abstract, original title, name of substance word, subject heading word, floating sub-heading word, keyword heading word, organism supplementary concept word, protocol supplementary concept word, rare disease supplementary concept word, unique identifier, synonyms]	**3**
4	PFPS.mp.	**533**
5	anterior knee pain.mp.	**2044**
6	Chondromalacia Patellae/	**96**
7	Sinding larsen johansson.mp.	**43**
8	runner* knee.mp.	**29**
9	plica syndrome.mp.	**119**
10	1 or 2 or 3 or 4 or 5 or 6 or 7 or 8 or 9	**4228**
11	Consensus/	**18440**
12	Consensus Development Conference/	**12306**
13	Consensus Development Conference, NIH/	**801**
14	(Consensus adj3 (statement or paper)).mp. [mp=title, abstract, original title, name of substance word, subject heading word, floating sub-heading word, keyword heading word, organism supplementary concept word, protocol supplementary concept word, rare disease supplementary concept word, unique identifier, synonyms]	**7476**
15	((position or policy) adj3 (statement or paper)).mp. [mp=title, abstract, original title, name of substance word, subject heading word, floating sub-heading word, keyword heading word, organism supplementary concept word, protocol supplementary concept word, rare disease supplementary concept word, unique identifier, synonyms]	**11880**
16	Practice Guideline/	**29792**
17	practice guideline.mp.	**35320**
18	Declaration.mp.	**9682**
19	11 or 12 or 13 or 14 or 15 or 16 or 17 or 18	**84269**

20	10 and 19	**14**

A.3 Search strategy for CINAHL via EBSCOhost

Friday, February 17, 2023 5:09:29 AM

#	Query	Limiters/Expanders	Last Run Via	Results
S20	S11 AND S19	Search modes - Boolean/Phrase	Interface - EBSCOhost Research Databases<br>Search Screen - Advanced Search<br>Database - CINAHL Complete	41
S19	S12 OR S13 OR S14 OR S15 OR S16 OR S17 OR S18	Search modes - Boolean/Phrase	Interface - EBSCOhost Research Databases<br>Search Screen - Advanced Search<br>Database - CINAHL Complete	106,124
S18	"policy statement"	Search modes - Boolean/Phrase	Interface - EBSCOhost Research Databases<br>Search Screen - Advanced Search<br>Database - CINAHL Complete	1,158
S17	"position paper"	Search modes - Boolean/Phrase	Interface - EBSCOhost Research Databases<br>Search Screen - Advanced Search<br>Database - CINAHL Complete	1,976
S16	"position statement"	Search modes - Boolean/Phrase	Interface - EBSCOhost Research Databases	3,569

			Search Screen - Advanced Search<br>Database - CINAHL Complete	
S15	(MH "Practice Guidelines")	Search modes - Boolean/Phrase	Interface - EBSCOhost Research Databases<br>Search Screen - Advanced Search<br>Database - CINAHL Complete	86,325
S14	"consensus statement"	Search modes - Boolean/Phrase	Interface - EBSCOhost Research Databases<br>Search Screen - Advanced Search<br>Database - CINAHL Complete	2,665
S13	(MH "Delphi Technique")	Search modes - Boolean/Phrase	Interface - EBSCOhost Research Databases<br>Search Screen - Advanced Search<br>Database - CINAHL Complete	7,854
S12	(MH "Consensus")	Search modes - Boolean/Phrase	Interface - EBSCOhost Research Databases<br>Search Screen - Advanced Search<br>Database - CINAHL Complete	7,272
S11	S1 OR S2 OR S3 OR S4 OR S5 OR S6 OR S7 OR S8 OR S9 OR S10	Search modes - Boolean/Phrase	Interface - EBSCOhost Research Databases<br>Search Screen - Advanced Search<br>Database - CINAHL Complete	5,741
S10	(MH "Plica Syndrome")	Search modes - Boolean/Phrase	Interface - EBSCOhost Research Databases<br>Search Screen - Advanced Search	18

		Database - CINAHL Complete		
S9	"runners knee"	Search modes - Boolean/Phrase<br>Search Screen - Advanced Search<br>Database - CINAHL Complete	Interface - EBSCOhost Research Databases	3
S8	(MH "Sinding-Larsen-Johansson Syndrome")	Search modes - Boolean/Phrase<br>Search Screen - Advanced Search<br>Database - CINAHL Complete	Interface - EBSCOhost Research Databases	18
S7	(MH "Chondromalacia Patella")	Search modes - Boolean/Phrase<br>Search Screen - Advanced Search<br>Database - CINAHL Complete	Interface - EBSCOhost Research Databases	99
S6	"anterior knee pain"	Search modes - Boolean/Phrase<br>Search Screen - Advanced Search<br>Database - CINAHL Complete	Interface - EBSCOhost Research Databases	1,101
S5	"PFPS"	Search modes - Boolean/Phrase<br>Search Screen - Advanced Search<br>Database - CINAHL Complete	Interface - EBSCOhost Research Databases	378
S4	"patellar femoral pain"	Search modes - Boolean/Phrase<br>Search Screen - Advanced Search<br>Database - CINAHL Complete	Interface - EBSCOhost Research Databases	3

S3	(MM "Patellofemoral Pain Syndrome")	Search modes - Boolean/Phrase<br>Search Screen - Advanced Search<br>Database - CINAHL Complete	Interface - EBSCOhost Research Databases	1,481
S2	patellofemoral pain	Search modes - Boolean/Phrase<br>Search Screen - Advanced Search<br>Database - CINAHL Complete	Interface - EBSCOhost Research Databases	2,292
S1	patellofemoral	Search modes - Boolean/Phrase<br>Search Screen - Advanced Search	Interface - EBSCOhost Research Databases	4,991

A.4 Search strategy for SPORTDiscus via EBSCOhost

Thursday, May 05, 2022 6:16:12 PM

#	Query	Limiters/Expanders	Last Run Via	Results
S18	(s10 or s11 or s12 or s13 or s14 or s15 or s16) AND (S9 AND S17)	Search modes - Boolean/Phrase	Interface - EBSCOhost Research Databases<br>Search Screen - Advanced Search<br>Database - CINAHL Complete	63

S17	s10 or s11 or s12 or s13 or s14 or s15 or s16	Search modes - Boolean/Phrase	Interface - EBSCOhost Research Databases<br>Search Screen - Advanced Search<br>Database - CINAHL Complete	159,590
S16	policy paper	Search modes - Boolean/Phrase	Interface - EBSCOhost Research Databases<br>Search Screen - Advanced Search<br>Database - CINAHL Complete	2,361
S15	position paper	Search modes - Boolean/Phrase	Interface - EBSCOhost Research Databases<br>Search Screen - Advanced Search<br>Database - CINAHL Complete	2,587
S14	position statement	Search modes - Boolean/Phrase	Interface - EBSCOhost Research Databases<br>Search Screen - Advanced Search<br>Database - CINAHL Complete	4,267
S13	practice guidelines	Search modes - Boolean/Phrase	Interface - EBSCOhost Research Databases<br>Search Screen - Advanced Search<br>Database - CINAHL Complete	101,391
S12	clinical practice guidelines	Search modes - Boolean/Phrase	Interface - EBSCOhost Research Databases<br>Search Screen - Advanced Search<br>Database - CINAHL Complete	12,550
S11	consensus statement	Search modes - Boolean/Phrase	Interface - EBSCOhost Research Databases<br>Search Screen - Advanced Search	4,051

			Database - CINAHL Complete	
S10	consensus	Search modes - Boolean/Phrase	Interface - EBSCOhost Research Databases<br>Search Screen - Advanced Search<br>Database - CINAHL Complete	55,743
S9	(S1 OR S2 OR S3 OR S4 OR S5 OR S6 OR S7 OR S8)	Search modes - Boolean/Phrase	Interface - EBSCOhost Research Databases<br>Search Screen - Advanced Search<br>Database - CINAHL Complete	6,099
S8	plica syndrome	Search modes - Boolean/Phrase	Interface - EBSCOhost Research Databases<br>Search Screen - Advanced Search	46

			Database - CINAHL Complete	
S7	runners knee	Search modes - Boolean/Phrase	Interface - EBSCOhost Research Databases<br>Search Screen - Advanced Search<br>Database - CINAHL Complete	196
S6	sinding-larsen-johansson syndrome	Search modes - Boolean/Phrase	Interface - EBSCOhost Research Databases<br>Search Screen - Advanced Search<br>Database - CINAHL Complete	23
S5	anterior knee pain	Search modes - Boolean/Phrase	Interface - EBSCOhost Research Databases	1,187

			Search Screen - Advanced Search<br>Database - CINAHL Complete	
S4	pfps	Search modes - Boolean/Phrase	Interface - EBSCOhost Research Databases<br>Search Screen - Advanced Search<br>Database - CINAHL Complete	915
S3	patellar femoral pain	Search modes - Boolean/Phrase	Interface - EBSCOhost Research Databases<br>Search Screen - Advanced Search<br>Database - CINAHL Complete	11
S2	patellofemoral pain	Search modes - Boolean/Phrase	Interface - EBSCOhost Research Databases<br>Search Screen - Advanced Search<br>Database - CINAHL Complete	2,292
S1	patellofemoral	Search modes - Boolean/Phrase	Interface - EBSCOhost Research Databases<br>Search Screen - Advanced Search<br>Database - CINAHL Complete	4,991

A.5 Search strategy for records from grey literature

Website Browsing Documentation:

**Date**	**Organization name** *Ex. Public Health Ontario*	**URL** *Ex. https://www.publichealthontario.ca/en/Pages/default.aspx*	**# of items screened (uploaded to citation management software)** *Ex. 3*
3-May-2022	OARSI (Osteoarthritis Research International) website	https://oarsi.org/ searching "consensus" and "guidelines"	1 (McAlindon et al, 2014)
3-May-2022	Patellofemoral.org	https://patellofemoral.org/about/	0
3-May-2022	ISAKOS	https://www.isakos.com/	0
3-May-2022	International Patellofemoral Research Network website	https://ipfrn.org/about/	0
3-May-2022	American Orthopaedic Society for Sports Medicine	https://www.sportsmed.org/	0

Website Searching Documentation:

**Date**	**Organization name & website URL**<br>*Ex. Healthy Ontario &*<br>*http://www.behealthyontario.com/*	**Search strategy(s)/ words searched including (if applicable) how items were selected.**<br>*Ex. Used Advanced Google site/domain search*<br>*Search strategies:*<br>*1) Wind turbines and sleep*<br>*2) Wind-powered electrical generators and sleep*<br>*Selection:*<br>*All items retrieved in with each search were reviewed for relevance by 1 reviewer.*	**# items retrieved/ search results**<br>*Ex. Search Results:*<br>*1) 18*<br>*2) 15*	**# of items screened (uploaded to citation management software)**<br>*Ex. Items Screened:*<br>*1) 3*<br>*2) 2*
3-May-2022	**Google Scholar (Canada)**	Patellofemoral AND consensus<br>Patellofemoral AND Guideline	1) 100 (first ten pages)<br>2) 100 (first ten pages)	1) 2 (Davis et al, 2010 & Powers et al 2012)<br>2) 2 (Bailey et al, 2021 & Geierlehner, et al 2020)
3-May-2022	**TRIP Database**	Patellofemoral consensus<br>Patellofemoral guidelines	1) 128<br>2) 52	1) 0<br>2) 0
3-May-2022	**Guidelines International Database (GIN)**	"Patellofemoral"<br>"Knee"<br>"Anterior knee"	1) 0<br>2) 23<br>3) 1<br>4) 0<br>5) 1<br>6) 0	1) 0<br>2) 0<br>3) 0<br>4) 0<br>5) 0<br>6) 0

		"Chondromalacia"<br><br>"Patella"<br><br>"PFPS"<br><br>"Runners Knee"	7) 0	7) 0
3-May-2022	**CPG Infobase**	"Patellofemoral"<br><br>"Knee"<br><br>"Anterior knee"<br><br>"Chondromalacia"<br><br>"Patella"<br><br>"PFPS"<br><br>"Runners Knee"	1) 0<br>2) 22<br>3) 0<br>4) 0<br>5) 0<br>6) 0<br>7) 0	1) 0<br>2) 0<br>3) 0<br>4) 0<br>5) 0<br>6) 0<br>7) 0
3-May-2022	**Clinical Key (1)**	"Patellofemoral"<br><br>"knee"<br><br>"Kneecap"<br><br>"Anterior knee"<br><br>"Chondromalacia"	1) 6<br>2) 57<br>3) 13<br>4) 3<br>5) 0<br>6) 13<br>7) 2<br>8) 4	1) 1 (Fox et al, 2018)<br>2) 0<br>3) 0<br>4) 0<br>5) 0<br>6) 0<br>7) 0<br>8) 0

		"Patella"<br><br>"PFPS"<br><br>"Runners Knee"		

Strategy 2. Grey Literature Database Search

**Date**	**Database name & URL**<br>*Ex. ClinicalTrials.Gov*<br>*&*<br>*https://clinicaltrials.gov/*	**Search strategy(s)/ words searched including (if applicable) how items were selected.**<br>*Ex. Search strategies:*<br>*1) Alzheimer's Disease and Canada.*<br>2) *Alzheimer Disease and Canada*<br>*Selection:*<br>*All results retrieved in the search were reviewed for relevance by 1 reviewer, 3 items were selected for screening.*	**# of items retrieved/ search results**<br>*Ex. Search Results:*<br>*1) 181*<br>*2) 3*	**# of items screened (uploaded to citation management software)**<br>*Ex. Items Screened:*<br>*1) 3*<br>*2) 0*
3-May-2022	Networked Digital Library of Theses and Dissertations (NDLTD)	1) Consensus AND patellofemoral<br>2) Guideline AND patellofemoral	1) 5<br>2) 6	1) 0<br>2) 0

	'Global ETD Search'			
3-May-2022	ProQuest Dissertations & Theses Global	1) Consensus AND patellofemoral<br>2) Guideline AND patellofemoral	1) 887<br>2) 1305	1) 0<br>2) 1 (Guanghua, Chinese CPG)
3-May-2022	Center for Research Libraries (CRL) Foreign Dissertation Database	1) Consensus AND patellofemoral<br>2) Guideline AND patellofemoral	1) 86<br>2) 27	1) 0<br>2) 0

Strategy 3. Search Engine Searching (Google.ca, DuckDuckGo.com)

**Date**	**Search engine**<br><br>*Ex. Google.ca*	**Search strategy(s) including how items were selected**<br><br>*Ex. Search strategies:*<br><br>*1) Wind turbines and sleep*<br><br>*2) Wind-powered electrical generators and sleep*<br><br>*Selection: Items were selected by scanning the first 100 results from each search*	**# of items screened (uploaded to citation management software)**<br><br>*Ex. Items Screened:*<br><br>*1) 3*<br>*2) 2*
3-May-2022	Google.ca	Intitle: 'Patellofemoral AND Consensus'<br><br>Intitle: 'Patellofemoral AND Guideline'	1) 9 (1 - Anand et al, 2022)<br>1) 9

Strategy 4. Contact Knowledge Experts

**Name of individual or listserv**	**Date contacted (follow up dates if necessary)**	**# of items recommended**	**# of items identified for full screening (uploaded to citation management software)**
Kay Crossley	July 2021	9	9 – IPRN consensus statements

This template was originally created by Jackie Stapleton and is based on the methods outlined in the article: Godin, K., Stapleton, J., Kirkpatrick, S. I., Hanning, R. M., & Leatherdale, S. T. (2015). Applying systematic review search methods to the grey literature: a case study examining guidelines for school-based breakfast programs in Canada. Systematic reviews, 4(1), 138. DOI: 10.1186/s13643-015-0125-0

Appendix B - Data extraction headings as utilized in Microsoft Excel.

Title	First Author	Year Published	Years since previous iteration	Aim of the consensus stated? (Rx recommendation, Definitions, Priority setting, etc)	No of panelists/experts	Experience of panelists (yrs)	**Expertise defined as…**	Was inclusion criteria for panelists explicit?	*If inclusion criteria was included please list here.*	What was the gender split?

How many countries were represented on the panel?	Were low / lower-middle income countries represented on the panel?	Did panelists remain same?	Participant groups included	**Was Stakeholder analysis completed?**	Were questions SR-informed?	Were the questions explicit?	What consensus method was reported?	Which method of consensus was used?

Was consensus level decided apriori?	What was the method or level of agreement set at?	Were dissenting opinions acknowledged and reported?	Scientometric impact to date?	Has the statement had any impact on Policy?	Funding / CoI

Appendix C - Excluded studies with reasons

Title	1st Author	Exclusion Reasons
Philadelphia Panel Evidenceâ€"Based Clinical Practice Guidelines on Selected Rehabilitation Interventions for Knee Pain	Albright, J.	No consensus process;
The 'Best Practice Guide to Conservative Management of Patellofemoral Pain': incorporating level 1 evidence with expert clinical reasoning	Barton, CJ	No consensus process
National Athletic Trainers' Association Position Statement: Management of Individuals With Patellofemoral Pain	Bolgla, LA	No consensus process (authors confirmed)
Patellofemoral arthroplasty in the athlete	Farr, J.	No consensus process
Patellofemoral pain syndrome: a review and guidelines for treatment	Juhn, MS.	No consensus process
Revision of the KNGF Guideline Osteoarthritis of the Hip and Knee	Peter, WFH.	No consensus process
Patellofemoral Instability: A Consensus Statement From the AOSSM/PFF Patellofemoral Instability Workshop	Post, WR	Focus is on patellofemoral instability
Treatment of osteoarthritis of the knee (nonarthroplasty)	Richmond, J.	No consensus process
Patellofemoral disorders: A classification system and clinical guidelines for nonoperative rehabilitation	Wilk, KE.	No consensus process
Patellofemoral Pain: Clinical Practice Guidelines Linked to the International Classification of Functioning, Disability and Health From the Academy of Orthopaedic Physical Therapy of the American Physical Therapy Association	Willy, RW.	No consensus process
Clinical classification of patellofemoral pain syndrome: Guidelines for non-operative treatment	Witvrouw, E.	No consensus process
MANAGEMENT OF FIRST-TIME PATELLAR DISLOCATION: A SURVEY OF PRISM MEMBERS	Parikh SN	Patellofemoral instability focus
Development of a return to play checklist following patellar instability surgery: a Delphi-based consensus	White AE	Patellofemoral instability focus
Consensus guidelines for management of patellofemoral instability	Bailey MEA	Patellofemoral instability focus

Patellar Instability Management A Survey of the International Patellofemoral Study Group	Liu JN	Patellofemoral instability focus
Patellofemoral Instability: A Consensus Statement From the AOSSM/PFF Patellofemoral Instability Workshop	Post WR	Duplicate: Patellofemoral instability focus
Recent advances and future trends in patellofemoral instability	Anand, BS	No consensus & Patellofemoral instability focus
Patellofemoral Pain Syndrome: Proximal, Distal, and Local Factors—An International Research Retreat: April 30–May 2, 2009, Fells Point, Baltimore, MD	Davis, I.	Duplicate
Patellofemoral Pain: Proximal, Distal, and Local Factors—Second International Research Retreat, August 31–September 2, 2011, Ghent, Belgium	Powers, C.	Duplicate
ACR Appropriateness Criteria(®) Chronic Knee Pain	Fox, MG.	Duplicate
Prevailing disagreement in the treatment of complex patellar instability cases: an online expert survey of the AGA Knee–Patellofemoral Committee	Geierlehner, A.	Patellar instability focus
