# Supplementary material for: Consensus methods in patellofemoral pain: how rigorous are they? A scoping review
Source: Br J Sports Med. 2024 May 22;58(13):733–44. doi: 10.1136/bjsports-2023-107552 (PMC11228197; doi:10.1136/bjsports-2023-107552)
Supplement: Supplementary data [file bjsports-2023-107552supp002.pdf]

Table 2. Characteristics of included consensus statements

Title	First Author	Year Published	Years since previous iteration	Stated aim of the consensus process (E.g. treatment recommendation, develop definitions, priority setting exercise, etc)
Selected issues for the adolescent athlete and the team physician: A consensus statement	Herring(26)	2008	n/a	Help the team physician improve the care of the adolescent athlete by understanding the medical, musculoskeletal (shoulder/knee-**including PFP**/elbow/spine), and psychological issues (sport specialization) common in this age group
Patellofemoral Pain Syndrome: Proximal, Distal, and Local Factors—An International Research Retreat: April 30–May 2, 2009, Fells Point, Baltimore, MD	Davis(11)	2010	n/a	Unclear - presentation of research and some future research recommendations
Patellofemoral Pain: Proximal, Distal, and Local Factors—Second International Research Retreat, August 31–September 2, 2011, Ghent, Belgium	Powers(27)	2012	2	To understand the factors that contribute to the development and, consequently the treatment of PFP
Patellofemoral pain: consensus statement from the 3rd International Patellofemoral Pain Research Retreat held in Vancouver, September 2013	Witvrouw(28)	2014	2	Gain consensus for three specific areas:<br>1. The natural history of PFP and local (knee region) factors that influence PFP;<br>2. Trunk and distal factors that influence PFP; and<br>3. Innovations in rehabilitation for PFP
2014 OARSI guidelines for the non-surgical management of knee osteoarthritis	McAlindon (29)	2014	6	To develop concise, up-to-date, patient-focused, evidence-based, expert consensus guidelines for the management of knee osteoarthritis (OA) *including patellofemoral osteoarthritis*
2016 Patellofemoral pain consensus statement from the 4th International Patellofemoral Pain Research Retreat, Manchester. Part 1: Terminology,	Crossley(30)	2016a	2	To agree on: Terminology, Definitions, Clinical Examination, Natural History, and PROMs (patient reported outcome measures)

definitions, clinical examination, natural history, patellofemoral osteoarthritis and patient-reported outcome measures				
2016 Patellofemoral pain consensus statement from the 4th International Patellofemoral Pain Research Retreat, Manchester. Part 2: recommended physical interventions (exercise, taping, bracing, foot orthoses and combined interventions)	Crossley(31)	2016b	2	To agree on expert recommended physical interventions for PFP
Selected issues in injury and illness prevention and the team physician: A consensus statement	Herring(32)	2016	0	To help the team physician improve the care of the athlete by understanding and practicing methods of injury and illness prevention in specific sports medicine problems.
Evidence-based framework for a pathomechanical model of patellofemoral pain: 2017 patellofemoral pain consensus statement from the 4th International Patellofemoral Pain Research Retreat, Manchester, UK: part 3	Powers(33)	2017	2	To place known associated factors within the context of a patho-mechanical model of PFP
Female Athlete Issues for the Team Physician: A Consensus Statement--2017 Update	Herring(34)	2018	n/a	To improve the care of the female athletes by understanding select injuries and illnesses
International patellofemoral osteoarthritis consortium: Consensus statement on the diagnosis, burden, outcome measures, prognosis, risk factors and treatment	Van Middlekoop(35)	2018	n/a	Mixed consensus on: diagnosis; burden; PROMs; Prognosis; Risk Factors; and Treatment.<br><br>Adds in a priority setting process.
2018 Consensus statement on exercise therapy and physical interventions (orthoses, taping and manual therapy) to treat patellofemoral pain: recommendations from the 5th International Patellofemoral Pain	Collins(36)	2018	2	To agree on expert recommended physical interventions for PFP

Research Retreat, Gold Coast, Australia, 2017				
The Diagnosis and Therapy of Degenerative Knee Joint Disease: Expert Consensus from the Chinese Pain Medicine Panel	Huang(37)	2018	n/a	To formulate Chinese Pain Specialist Consensus on the diagnosis and treatment of degenerative knee osteoarthritis (DKOA)
ACR Appropriateness Criteria(®) Chronic Knee Pain	Fox(38)	2018	n/a	To provide clinicians with the best practices for ordering imaging examinations
Chinese Guidelines for Diagnosis and Treatment of Patellofemoral Osteoarthritis (2020 Edition)	Guanghua(39)	2020	n/a	Guidelines on the diagnosis and treatment of patellofemoral OA
An Expert Consensus Statement on the Management of Large Chondral and Osteochondral Defects in the Patellofemoral Joint	Chahla(40)	2020	na	Consensus on the functional anatomy, indications, donor graft considerations, surgical treatment, and rehabilitation of large chondral and osteochondral defects in the patellofemoral joint
2019 American College of Rheumatology/Arthritis Foundation Guideline for the Management of Osteoarthritis of the Hand, Hip, and Knee	Kolasinski(41)	2020	8	To develop an evidence-based guideline for the comprehensive management of osteoarthritis (OA) as a collaboration between the American College of Rheumatology (36) and the Arthritis Foundation, updating the 2012 ACR recommendations for the management of hand, hip, and knee OA.
Operative Treatment Options for Patellofemoral Arthritis: An Expert Recommendation of the AGA Patellofemoral Committee	Keshmiri(42)	2021	na	Consensus on therapy for different patellofemoral abnormalities in patients suffering from isolated patellofemoral arthritis.

Community-based rehabilitation implementation framework to address PFP amongst runners in under-resourced communities: Delphi consensus	Kunene(43)	2021	na	To develop a community-based rehabilitation implementation framework for PFP in runners from under-resourced communities
REPORT-PFP: a consensus from the International Patellofemoral Research Network to improve REPORTing of quantitative PatelloFemoral Pain studies	Barton(44)	2021	3	Consensus statement and associated checklist provides standards for REPORTing of quantitative PatelloFemoral Pain (REPORT-PFP) research to enhance clinical translation and evidence synthesis, and support clinician engagement with research and data collection
Expert consensus on surgical treatment of patellofemoral osteoarthritis	Guanghua(45)	2021	na	To summarize the latest research progress in the surgical treatment of patellofemoral osteoarthritis, and refer to the latest domestic and foreign guidelines and consensus
Developing Clinical and Research Priorities for Pain and Psychological Features in People With Patellofemoral Pain: An International Consensus Process With Health Care Professionals	Vicenzino(46)	2022	3	The objective of this consensus development process was to decide clinical and research priorities for pain features and psychological factors in persons with patellofemoral pain.
