# Supplementary material for: Consensus methods in patellofemoral pain: how rigorous are they? A scoping review
Source: Br J Sports Med. 2024 May 22;58(13):733–44. doi: 10.1136/bjsports-2023-107552 (PMC11228197; doi:10.1136/bjsports-2023-107552)
Supplement: Supplementary data [file bjsports-2023-107552supp003.pdf]

Table 3. Representativeness of the panels

First Author	*No. panelists*	*Experience / definition of expertise*	*Inclusion criteria for panelists*	*Sex split*	*No. countries - represented*	*low / lower-middle income countries represented*	*Participant groups included*
Herring (2008)	11	**n/a**	**Yes* (see key)**	7 male: 4 female	1 - all situated in the USA	No	10 MD***, 1 Doctor of Osteopathy
Davis (2010)	Unclear	n/a	Unclear - Had to be part of the PFP Research Retreat - unclear how involvement was facilitated	unclear	10 - (Australia, Belgium, Brazil, Canada, Israel, Italy, Netherlands, Singapore, UK, USA.)	No	Unclear (scientists, researchers, clinicians)
Powers (2012)	Unclear	n/a	Unclear - Had to be part of the PFP Research Retreat - unclear how	unclear	9 - not reported which countries (Lead author from USA)	Unclear	Unclear (scientists, researchers, clinicians)

			involvement was facilitated				
Witvrouw (2014)	Not reported	n/a	Unclear - Had to be part of the PFP Research Retreat - unclear how involvement was facilitated	unclear	not reported (Authors from Qatar, Belgium, UK, USA, and Australia)	Unclear	Unclear (scientists, researchers, clinicians)
McAlindon (2014)	13	n/a	Yes - "expert panel was composed of 13 voting members and a patient advocate. This group was selected for its diverse expertise and experience in OA management"	10 male: 3 female	10 (USA, UK, France, Netherlands, Belgium, Sweden, Denmark, Australia, Japan, and Canada)	No	Medical disciplines (primary care, rheumatology, orthopedics, physical therapy, physical medicine and rehabilitation, and evidence-based medicine) **& 1 patient**

Crossley (2016a)	Not reported	n/a	No - mention of 'whole group discussion to facilitate consensus'	unclear	not reported (authors from Australia, UK, USA, and Canada)	Unclear	Unclear
Crossley (2016b)	35	n/a	Unclear - Had to be part of the PFP Research Retreat - unclear how involvement was facilitated	unclear	not reported (authors from Australia, Netherlands, UK and Denmark)	Unclear	Six groups: Physiotherapists; MD; Podiatrists; Biomechanists; Epidemiologists; and Sports Therapists
Herring (2016)	12	n/a	**Yes* (see key)**	10 male:2 female	1 - all situated in the US	No	10 MD, 2 Doctors of Osteopathy
Powers (2017)	Not reported	n/a	Unclear - Had to be part of the PFP Research Retreat - unclear how involvement was facilitated	unclear	not reported (authors from USA, Belgium, Australia)	Unclear	Unclear

Herring (2018)	10	n/a	**Yes* (see key)**	4 male:6 female	2 - all situated in the USA & Canada	No	8 MD, 2 Doctors of Osteopathy
Van Middlekoop (2018)	Unclear - 17 authors	n/a	No - 'invited based on scientific publications or interest in the field'	Unclear - authorship 8 male : 9 female	Unclear - authors from Netherlands, Australia, UK and USA	No	Physical Therapy; Rheumatology; Orthopaedics; Radiology; Epidemiology; Human Movement Science; General Health Science
Collins (2018)	41	n/a - justification that all were active researchers in the field	No - had to be part of the research retreat. Unclear how panel members were selected down from original numbers.	Unclear - authorship 6 male : 6 female	Unclear - authors from Australia, Netherlands, UK, Denmark, USA, Canada, and Brazil.	Unclear	Physical Therapy; Athletic Trainers; Podiatrists; Biomechanists; Biomechanical Engineering; and Human Movement Science

Huang (2018)	15	n/a	No	Unclear	1 - all situated in China	No	Unclear
Fox (2018)	17	n/a	**Yes** (See key)**	8 male: 9 female	1 - USA-only	No	17 MDs (radiology experts plus others)
Guanghua (2020)	30	n/a	No	Unclear	1 - all situated in China	No	unclear - specialties of panel is not listed.
Chahla (2020)	28	n/a	Experts had a clinical practice that utilizes cartilage restorative procedures for the patellofemoral joint (minimum of 30 cartilage cases per year), and had frequently published and/or lectured on the topic (>10 publications on patellofemoral	26 male: 2 female	2 – USA & Canada	No	Surgeons-only

			chondral injuries). Experts were part of a previously established group (2016) of osteochondral allograft experts (MOCA group).				
Kolasinski (2020)	15	n/a	No. Expressions of interest are invited by the American College of Rheumatology. *However, how panel were recruited and against what metrics is not reported.*	8 male : 7 female	2. USA & Canada	No	10 Rheumatology/MDs; 1 Internist-MD; 1 Physical therapist; 1 Occupational therapist; **1 patient.**
Keshmiri (2021)	13	n/a	No. AGA (*AGA not defined in the paper or*	12 male: 1 female	3 – Germany, Austria and Switzerland	No	Surgeons-only

			online) patellofemoral committee members.				
Kunene (2021)	19	5-10 years =3; 11-20 years =10; >20 years =6<br><br>**Defined expertise** - Min 5-years post qualification	Yes. Clinicians who were experienced in working with runners in marginalized communities. The participants had to have at least 5 years of experience in the treatment and rehabilitation of running-related injuries including PFP.	**10 female**: 9 male	2 - South Africa (15) and UK (4)	No	Physician(3); Physical therapist (6); Sports Therapist (2); Biokineticist (2); Podiatrist (2); Dietician (2); Psychologist (2)
Barton (2021)	24 (2015) and then 51 (2019)	24% of final voting panel <4 years clinical	Yes. Must have been a previous attendee of the International PFP research retreat.	Unclear*	10 countries (Australia=7; Brazil=7; Canada=3; Denmark=3;	Yes - 1 member from India	Multiple layers Round 1: Physiotherapists - 19; Athletic Trainers - 1;

		experience; 30% >16 years clinical experience			India=1; Italy=2; Kuwait=1; The Netherlands=2; UK=9; USA=16).		Sports MD - 2; Biomechanist -1; Other - 1.<br><br>Round 2-NA<br><br>Round 3 - 51 (Physiotherapists 26, Athletic Trainers -7, MD -2).<br><br>Final voting section unclear on professional affiliations.
Guanghua (2021)	35	n/a	No - had to be an orthopaedic 'expert'	Unclear	1 – China-only	No	Orthopaedic-surgeon specialists only

Vicenzino (2022)	71 = 35 for **survey** (plus 36 patients)<br><br>**In-person** 20	Involvement with PFP ranging from 0 to >16 years*<br>**Defined expertise** in years of experience or exposure to PFP patients	Yes - Experts were identified through the International Patellofemoral Research Network, www.expertscape.com, peer-reviewed publications, and screening abstract submissions to the 2019 International Patellofemoral Research Retreat (Milwaukee, WI).	**Survey**: healthcare workers N=35 (20 male:15 female); and patients (12 male:24 female)<br><br>**In-person:** (11 male: 9 female)	9 in health professions survey (Australia, Canada, Spain, Brazil, Denmark, Ireland, Netherlands, UK and USA)<br>4 patient survey (78% UK, 11% Denmark, 8% Australia and 3% Ireland)<br><br>8 at the in-person process (Australia = 6; Brazil = 2; Denmark = 1; Ireland = 1; The Netherlands	No	Survey round: Physiotherapists (12), Athletic trainers(3), Orthopaedic Surgeon(3), Sports Medicine MD(3), Exercise Scientist (1), Biomechanist(1), Psychologist(1), Radiologist(1), Other1)<br><br>**In-person:** Physiotherapists (12); Athletic trainers (3); Sports Medicine MD (1); Exercise Scientist (1); Biomechanist (1);

	= 1; UK = 3; US = 5; Italy =1)	Psychologist (1); Other (1)

Key:

*Panel is made up from 2 nominated representatives from each of American Academy of Family Physicians, American Academy of Orthopaedic Surgeons, ACSM, American Medical Society for Sports Medicine, American Orthopaedic Society for Sports Medicine and American Osteopathic Academy of Sports Medicine. Representatives are chosen by their organization based on their experience as team physicians with expertise in the topic area.

** Available on American College of Radiology (ACR) website - "Following regulatory requirements, we survey panel members on their skills and expertise to ensure that panels include expertise in the clinical topic, primary care medicine, medical imaging, statistics, and clinical trial design. Panel members' expertise is determined using self-attestation and calculated by the amount of education, training, and experience the member reports for that skill area"

***MD = Medical Doctor
