# Supplementary material for: Consensus methods in patellofemoral pain: how rigorous are they? A scoping review
Source: Br J Sports Med. 2024 May 22;58(13):733–44. doi: 10.1136/bjsports-2023-107552 (PMC11228197; doi:10.1136/bjsports-2023-107552)
Supplement: Supplementary data [file bjsports-2023-107552supp004.pdf]

Table 4. Methods used for measuring, and/or facilitating consensus among panelists.

First Author	*What consensus method was reported?*	*Which method of consensus was used?*	*Was consensus level decided apriori?*	*What was the method or level of agreement set at?*	*Were dissenting opinions acknowledged and reported?*
Herring (2008)	None	Informal - iterative development over written rounds and in-person meeting	No	Unanimous agreement amongst invited panel - assumed *not* measured	No
Davis (2010)	None	Consensus conference – informal	No	Unclear	No
Powers (2012)	None	Consensus conference – informal	No	Unclear	No
Witvrouw (2014)	None	Consensus conference – informal	No	Unclear	No
McAlindon (2014)	RAND-UCLA & Delphi	RAND-UCLA	Yes	RAND method (Median score of greater than 7 using a 9-point Likert, with less than 1/3 of panelists also not falling in the 1-3 range indicating a large disagreement)	yes - as part of the RAND-UCLA method
Crossley (2016a)	None	Consensus conference – informal	No	Unclear	No
Crossley (2016b)	Unclear	Voting - report “OARSI method” - assessed as	Yes	Had to be rated 'appropriate' (7-9 on a 10-point Likert) on	No

		a modified version of RAND/UCLA		average (median) *AND* consistent with evidence	
Herring (2016)	None	Informal - iterative development over written rounds and in-person meeting	No	Unanimous agreement amongst invited panel - assumed *not* measured	No
Powers (2017)	None	Informal - not reported fully	No	Unclear	No
Herring (2018)	None	Informal - iterative development over written rounds and in-person meeting	No	Unanimous agreement amongst invited panel - assumed *not* measured	No
Van Middlekoop (2018)	Numerical Rating Scale 0-10	Unclear - no report of consensus process NRS 0-10 used for priority setting	Yes	For priority setting part only, consensus was >7.5 out of 10 on numerical rating scale	No
Collins (2018)	10 point Likert scale - median score must be between 7-9	Unclear - median level of agreement but also had to agree with evidence	Yes - level of certainty	median agreement between 7-9	no - outliers were given on bar graphs, not discussed
Huang (2018)	None	n/a	Unclear	Unclear	No
Fox (2018)	RAND-UCLA	RAND-UCLA	Yes	median agreement between 7-9	not explicit but is part of RAND method
Guanghua (2020)	Delphi	Delphi - unclear whether also involved in-person round	Unclear	Unclear	No
Chahla (2020)	Delphi	Modified Delphi*	Yes	over 75% of respondents agreed and fewer than 20%	No

				disagreed in the final voting round	
Kolasinski (2020)	Unclear	Unclear - face to face voting over 2-days reported.	Yes	> 70% agreement	No
Keshmiri (2021)	Delphi	Modified Delphi	No	n/a – did not seek to measure consensus, just reported level of agreement with statements	No
Kunene (2021)	Delphi	Modified - Delphi	Yes	> 70% agreement	No
Barton (2021)	Delphi	Modified Delphi mixed with Priority setting process (see earlier work of the same group)	Yes for stage 3	> 70% agreement	No
Guanghua (2021)	Unclear	Unclear	Unclear	Unclear	No
Vicenzino (2022)	Survey plus in person meeting	Survey plus in person meeting	Yes	> 70% agreement on survey and paper based votes (single round)	No
